# Environmental Exposure-Induced Epigenetic Modifications and Adverse Pregnancy Outcomes: A Systematic Review

**DOI:** 10.7759/cureus.109320

**Published:** 2026-05-20

**Authors:** Raj Kishor Sharma, Usha Kumari

**Affiliations:** 1 Microbiology, Indira Gandhi Institute of Medical Sciences (IGIMS), Patna, IND; 2 Biochemistry, Indira Gandhi Institute of Medical Sciences (IGIMS), Patna, IND

**Keywords:** endocrine disruptors, epigenetics, fetal development, pregnancy, reproductive health

## Abstract

Prenatal environmental exposures may alter epigenetic regulation, influencing fetal development and pregnancy outcomes. The aim of the study was to systematically review original studies examining associations between environmental exposures, epigenetic modifications, and pregnancy-related disorders. A systematic search of PubMed, ScienceDirect, Cochrane Library, and Google Scholar identified studies evaluating prenatal exposures, epigenetic markers, and clinical outcomes. Study quality was assessed using the Joanna Briggs Institute criteria. Thirty-four studies (2009-2024) were included. Prenatal exposure to air pollutants, heavy metals, endocrine disruptors, and tobacco smoke was associated with global and gene-specific DNA methylation changes, altered miRNA profiles, and histone modifications in placental and cord-blood tissues. These epigenetic alterations were frequently linked to preterm birth, low birth weight, stillbirth, gestational diabetes, and hypertensive disorders of pregnancy. Environmental exposures during pregnancy are consistently associated with measurable epigenetic changes that parallel adverse birth outcomes, supporting their potential role as mechanistic mediators and biomarkers of risk.

## Introduction and background

Environmental pollution during pregnancy is a major public health concern, particularly because fetal development represents a highly vulnerable window of exposure [1-3]. The intrauterine environment plays a decisive role in shaping long-term health trajectories [4,5]. Adverse birth outcomes, including preterm birth, low birth weight (<2,500 g), small-for-gestational-age (SGA), stillbirth, and preeclampsia, remain leading contributors to neonatal morbidity and mortality worldwide and align with the Developmental Origins of Health and Disease (DOHaD) framework linking early-life exposures to adult disease [1,6]. Environmental exposures during pregnancy may influence fetal development through epigenetic mechanisms, including DNA methylation, histone modification, and non-coding RNA regulation, without altering the underlying DNA sequence. These environmentally induced epigenetic alterations may affect placental function, fetal growth, immune regulation, and long-term offspring health.

Emerging evidence indicates that environmentally induced epigenetic dysregulation at the maternal-fetal interface is a central mechanism connecting prenatal exposures to adverse perinatal outcomes [7,8]. Epidemiological studies have associated exposure to ambient air pollution, environmental tobacco smoke, heavy metals, endocrine-disrupting chemicals (EDCs), water contaminants, radiation, and persistent organic pollutants with pregnancy loss, impaired fetal growth, preterm birth, and hypertensive disorders of pregnancy [9-13]. Meta-analytic data further support associations between prenatal PM₂.₅ exposure, stillbirth, and reduced birth weight [14-16]. Fine particulate matter can cross the alveolar-blood barrier, triggering systemic inflammation and oxidative stress that may impair placental function. Beyond classical toxicological exposures, emerging gestational exposome frameworks also recognize the contribution of psychosocial stress, maternal mental health, climate-related exposures, and combined environmental stressors to fetal programming. These exposures may converge through shared biological pathways involving oxidative stress, inflammation, endocrine disruption, placental dysfunction, and neuroendocrine signaling, potentially influencing fetal neurodevelopment and long-term offspring health [17-19].

Epigenetic mechanisms, including DNA methylation, histone modifications, genomic imprinting, and non-coding RNAs, regulate gene expression without altering the DNA sequence. The placenta undergoes dynamic epigenetic remodeling to coordinate immune tolerance, endocrine signaling, and nutrient exchange. Prenatal NO₂ and O₃ exposures have been linked to differentially methylated regions (DMRs) in RNF39 and CYP2E1 in the placenta and cord blood [20], while maternal PM₂.₅ exposure has been associated with global hypomethylation of repetitive elements such as LINE-1 and Alu [21]. Additionally, early gestational exposure to elevated NOₓ has been associated with epigenetic age deceleration in placentas from late-onset preeclampsia cases [15]. These findings suggest that environmentally induced epigenetic alterations may parallel, and potentially mediate, adverse birth outcomes.

Despite increasing evidence, substantial heterogeneity exists in exposure assessment, tissue selection, epigenetic endpoints, and outcome definitions across studies. To date, no comprehensive synthesis has systematically integrated human epidemiological data linking diverse prenatal environmental exposures with epigenetic modifications and pregnancy-related disorders. Therefore, this systematic review aims to summarize original research examining how environmental exposures, including air pollutants, heavy metals, endocrine disruptors, and tobacco smoke, induce epigenetic alterations and how these changes relate to pregnancy complications and fetal outcomes.

## Review

Materials and methods

This systematic review was conducted in accordance with Preferred Reporting Items for Systematic reviews and Meta-Analyses (PRISMA) guidelines [22].

Search Strategy

A comprehensive search of PubMed, the Cochrane Library, ScienceDirect, and Google Scholar was performed through June 2025, with no restrictions on country or publication date. Search terms combined concepts related to environmental exposures (e.g., pollution, endocrine disruptors, heavy metals), epigenetic modifications (DNA methylation, histone modification, non-coding RNA), and pregnancy outcomes (e.g., preterm birth, preeclampsia, gestational diabetes). Only original research articles were considered; editorials, conference abstracts, guidelines, and reviews were excluded.

The review followed a PECO framework: females of reproductive age (P), exposure to environmental pollutants or endocrine disruptors (E), comparator/control groups (C), and pregnancy-related outcomes including gestational diabetes, hypertensive disorders, stillbirth, abortion, and reproductive health conditions (O).

Although this review was not prospectively registered in PROSPERO, PRISMA methodology and predefined eligibility criteria were followed to improve methodological transparency.

Study Selection and Eligibility

After duplicate removal, titles and abstracts were screened, followed by independent full-text review. Eligible studies included cohort, case-control, or cross-sectional designs reporting associations between prenatal environmental exposures, epigenetic markers (e.g., DNA methylation, histone modifications, miRNA expression), and pregnancy outcomes (e.g., preterm birth, low birth weight, preeclampsia).

Studies were excluded if they lacked epigenetic or clinical outcome data, were non-quantitative, duplicated data, or were case reports, commentaries, editorials, or meta-analyses. Reference lists of relevant reviews were screened to identify additional studies.

Data Extraction and Quality Assessment

Due to substantial methodological heterogeneity across exposures, tissues, epigenetic endpoints, and outcome definitions, a narrative synthesis approach was considered more appropriate than formal meta-analysis. Data extracted included study characteristics, exposure type, epigenetic endpoints, and pregnancy outcomes. Two reviewers independently performed data extraction and risk-of-bias assessment, with discrepancies resolved by consensus. Study quality was evaluated using the Joanna Briggs Institute (JBI) Critical Appraisal Checklist [23], and studies were categorized as low, moderate, or high risk of bias based on predefined scoring thresholds.

Results

Identification and Description of Studies

**Figure 1:**
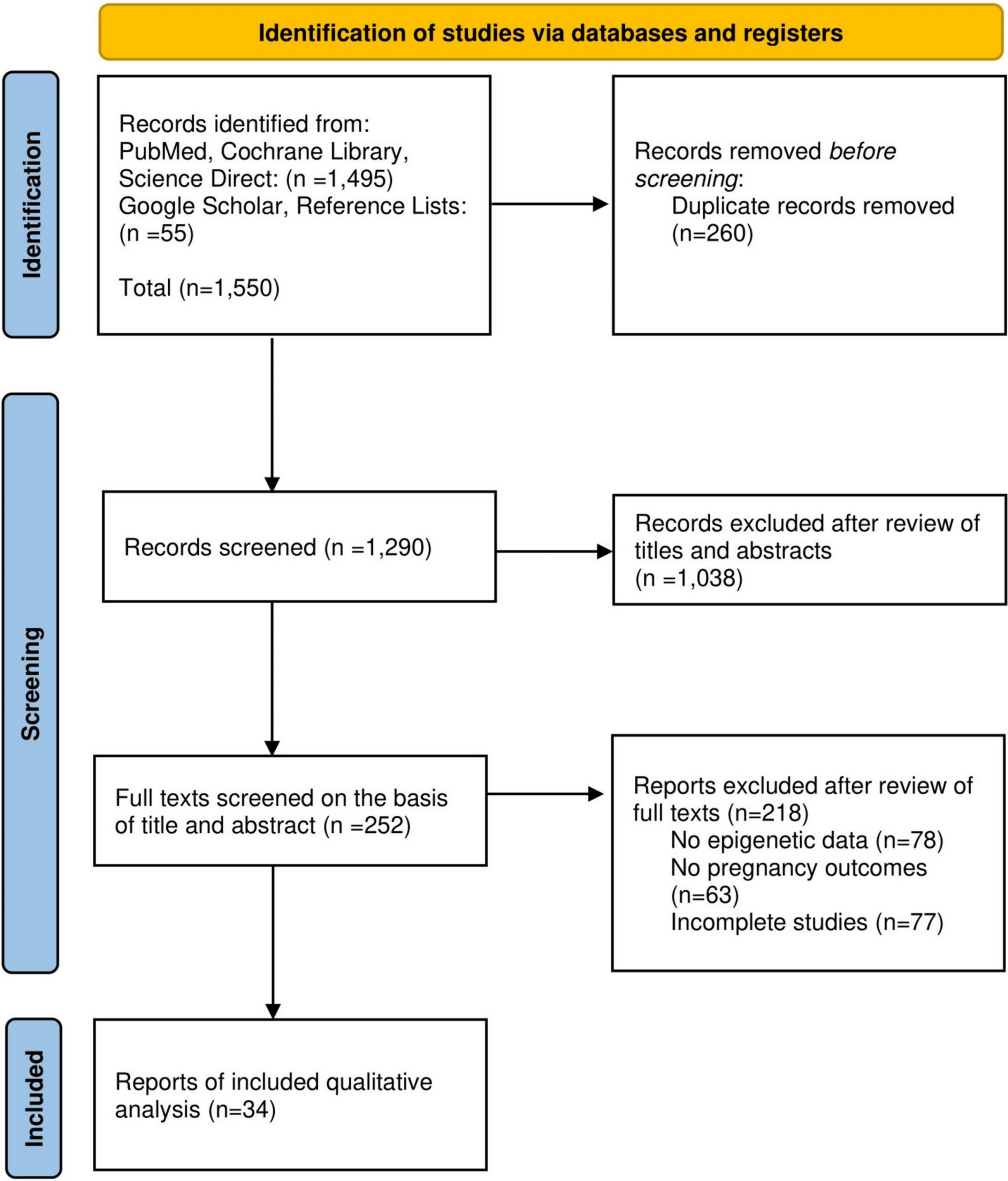
PRISMA flow diagram. PRISMA: Preferred Reporting Items for Systematic reviews and Meta-Analyses

A total of 34 studies were included in the final qualitative analysis (Figure 1), with key characteristics summarized in Table 1.

**Table 1:** Key characteristics of the included studies. DEHP: Di-(2-ethylhexyl) phthalate; DMRs: differentially methylated regions; SGA: small-for-gestational age; GDM: gestational diabetes mellitus; PTB: preterm birth

Study	Country	Sample Size	Study Design	Exposure Domain	Epigenetic Marker	Outcome	Key Findings
Chen et al., (2018) [24]	Taiwan	64 mother–infant pairs	Prospective birth cohort	Maternal DEHP metabolites	Cord-blood genome-wide DNA methylation (Illumina 450K)	Differential CpG methylation	25 CpG sites (FDR < 0.05) correlated with DEHP; enriched in androgen/estrogen-response and spermatogenesis genes.
Janssen et al., (2013) [21]	Belgium	240 mother–newborn pairs	Prospective birth cohort	Maternal PM₂.₅ (3rd trimester)	Global placental LINE-1 & Alu methylation (pyrosequencing)	Percent methylation of repeats	Early prenatal exposure to particle air pollution, especially throughout the crucial stages of implantation, was linked to a decreased level of placental global DNA methylation.
You et al., (2024) [19]	South Korea	454 pregnant women	Prospective cohort	Trimester-specific maternal PM₂.₅	Cord-blood mtDNA copy number; OXPHOS enzyme activity	Preterm birth; GDM; mitochondrial dysfunction	High PM2.5 exposure during pregnancy may dysregulate genes linked to PTB and raise the risk of GDM and PTB.
Ivorra et al., (2015) [25]	Spain	20 mother–newborn pairs	Case–control cohort	Maternal smoking	Cord-blood methylation (450K EWAS; pyrosequencing of AHRR, CYP1A1)	Differential CpG methylation patterns	In utero tobacco exposure may alter the epigenome, contributing to global DNA hypomethylation.
Feil et al., (2023) [26]	South Africa	142 mother–child pairs	Prospective cohort	Prenatal indoor PM₁₀ (2nd trimester personal monitoring)	Cord-blood methylation (450K & EPIC arrays)	Cognitive development at 2 yrs	DNAm may mediate the association between prenatal PM10 exposure and cognitive neurodevelopment
Abraham et al., (2018) [27]	France	668 placentas	Prospective mother–child cohort	Maternal NO₂, PM₁₀, ambient temperature & humidity	Placental genome‐wide DNA methylation (Illumina 450K)	Differential CpG methylation and DMRs	Air pollutant exposure led to placental gene methylation and preeclampsia.
Weyde et al., (2021) [28]	Norway	631 mother–child pairs	Prospective birth cohort	Gestational blood levels of 5 toxic metals & 7 essential elements	Maternal & cord-blood global 5mC & 5hmC (LC-MS)	Global DNA methylation	Global DNA methylation in expectant mothers and their newborn offspring was discovered to be associated with gestational levels of a number of toxic metals.
Pilsner et al., (2009) [29]	Mexico City	103 mother–infant pairs	Cross-sectional within a prospective cohort	Maternal bone lead (patella & tibia by K-XRF)	Cord-blood LINE-1 & Alu methylation (pyrosequencing)	Global repeat methylation (% 5mC)	Prenatal lead exposure is inversely associated with genomic DNA methylation in cord blood.
Herrera-Moreno et al., (2022) [30]	Mexico City	507 mother–child pairs	Prospective birth cohort	Maternal blood Pb (2nd, 3rd trimester, delivery) & bone Pb (postpartum)	Cord-blood leukocyte telomere length (qPCR)	Telomere length & DNAm age biomarkers	No significant associations between prenatal lead (blood or bone) and telomere length or DNA methylation age in newborns were found
Cardenas et al., (2017) [31]	USA	306 mother–child pairs	Prospective pre-birth cohort	Maternal 2nd-trimester RBC-Hg concentration	Cord-blood & childhood blood	Global %-5mC & %-5hmC at birth, early & mid-childhood	Our findings show the possible malleability of epigenetic changes linked to fetal mercury exposure.
Yang et al., (2018) [32]	China	95,354 births	Population-based prospective birth cohort	Daily mean PM₂.₅, PM₁₀, SO₂, NO₂, CO, O₃ (trimester‐specific & whole pregnancy)	Not applicable	Stillbirth	PM₂, PM₁₀, SO₂, CO, and O₃ all raise the chance of stillbirth in mothers. The most vulnerable periods seem to be the second and third trimesters. These results support the idea that air pollution affects prenatal development.
Quraishi et al., (2022) [33]	USA	2,099 mother–child pairs	Prospective pre-birth cohorts	Prenatal PM₂.₅	Not applicable	Birthweight; preterm birth; SGA	Lower birthweight was linked to higher PM₂.₅ during pregnancy. Families with lower family incomes and male infants saw greater effects.
Chen et al., (2023) [34]	China	56,905 singleton pregnancies	Prospective cohort	Preconception to delivery ozone (8-h max daily); ambient temperature as effect modifier	Not applicable	Preterm birth; LBW; LGA	High ambient temperature (>75th percentile) strengthened O₃ associations: 2nd-trimester LBW; full-pregnancy LGA; effects were null or weaker at lower temperatures.
Chen et al., (2018) [35]	Australia	Not specified	Population-based retrospective birth cohort	Daily mean PM₂.₅, SO₂, NO₂, O₃ (trimester-specific & whole pregnancy)	Not applicable	Preterm birth; low birth weight	Pregnancy-related low-level exposures to PM₂, SO₂, NO₂, and O₃ were associated with increased risks of PTB and LBW. The most vulnerable period was the third trimester, and relationships were most pronounced in low to moderate ambient temperatures.
Liu et al., (2019) [36]	China	3,550 women (1,784 cases: 687 PTB, 1,097 LBW; 1,766 healthy controls)	Case–control	PM₂.₅, PM₁₀, SO₂, NO₂, CO, O₃ (daily averages; 1st/3rd trimester for PTB; first/last month for LBW)	Not applicable	Preterm birth; low birth weight	This study provides additional evidence of the links between low birth weight and preterm delivery and air pollution. Reducing or avoiding exposure to air pollution is suggested for expectant mothers, particularly during the early and late stages of pregnancy.
Yorifuji et al., (2015) [37]	Japan	44,109 term singletons	Nationwide population-based longitudinal cohort	Municipal-level suspended particulate matter (SPM), NO₂, SO₂ (9-month average; trimester-specific)	Not applicable	Term low birth weight	Ambient air pollution increases the risk of term low birth weight in a nationally representative sample in Japan.
Kim et al., (2019) [17]	Republic of Korea	1,742,183 singleton births	Nationwide registry‐based birth cohort	Mean pregnancy PM₁₀ (μg/m³) categorized by WHO interim target	Not applicable	Preterm birth; term low birth weight (LBW)	PM10 exposure > 70 µg/m3 was associated with preterm births.
Johnson et al., (2016) [38]	USA	258,294 singleton births	Population-based cohort	Ambient PM₂.₅ and NO₂ (first, second, third trimesters; spatial & temporal metrics from NYCCAS and monitors)	Not applicable	Spontaneous preterm birth (<37 wk)	Neither PM₂.₅ nor NO₂ was positively associated with spontaneous preterm birth
Siddika et al., (2020) [39]	Finland	2,427 mother–infant pairs	Population-based cohort	Short-term (week before delivery) and long-term (entire pregnancy) PM₂.₅, PM₁₀, NO₂, SO₂, CO, O₃ (LUR & dispersion)	Not applicable	Preterm birth	Exposure to low-level ambient air pollution may trigger preterm birth
Adibi et al., (2017) [10]	USA	180 placentas (358 women enrolled)	Prospective urban birth cohort	Late‐pregnancy urinary phthalate monoesters (MnBP, MBzP, MiBP, MEOHP)	Placental mRNA levels of 8 candidate genes (qPCR)	Gene expression & birth outcomes	Sex‐specific associations between phthalate metabolites and placental mRNAs (CGA, PPARG, CYP19A1, CYP11A1), stronger effects in male placentas and modified by gestational diabetes mellitus.
Arbuckle et al., (2014) [11]	Canada	2,000 first‐trimester women	Prospective cohort	First‐trimester spot urine total BPA (GC–MS/MS) and 11 phthalate metabolites (LC–MS/MS); SG‐corrected	Not applicable	Urinary BPA & phthalate levels	Geometric mean urinary BPA = 0.80 µg/L; 88% detections. Highest phthalates: MEP GM 32.02 µg/L, MnBP GM 11.59 µg/L. Levels varied by maternal age, smoking, fasting status, nativity, income, and education.
Ladd‐Acosta et al., (2019) [20]	USA	133 placentas; 175 cord‐blood samples	Prospective EWAS	Prenatal NO₂ and O₃ at maternal residence (trimester‐specific & full‐pregnancy averages)	Placental and cord‐blood genome‐wide DNA methylation (Illumina 450K)	Global Δβ by context; DMR mapping	NO₂ and O₃ exposures were associated with differential methylation across both placenta and cord blood, including DMRs in RNF39 and CYP2E1, with sex‐specific patterns.
Straughen et al., (2024) [40]	USA	64 pregnant African American women	Prospective urban birth cohort	Personal 48-h passive air sampling of benzene, toluene, ethylbenzene, and xylene (BTEX)	Maternal whole‐blood genome‐wide DNA methylation (Illumina EPIC)	Differential CpG methylation: preterm birth risk	Changes in DNA methylation are linked to BTEX exposure, and there is a general tendency that increased BTEX exposure is linked to hypomethylation, which raises the risk of preterm delivery.
Martínez‐Ibarra et al., (2019) [12]	Mexico	40 pregnant women (18 GDM; 22 controls)	Clinic‐based case–control	Urinary phthalate metabolites (MBP, MiBP, MBzP, MEHP) and BPA (2nd‐trimester)	Serum miRNAs (miR-9-5p, miR-16-5p, miR-29a-3p, miR-330-3p)	miRNA expression profiles	miR-9-5p, miR-29a-3p, miR-330-3p were elevated in GDM patients vs controls; MBzP positively correlated with miR-16-5p, MEHP with miR-29a-3p, and MBP/MiBP inversely with miR-29a-3p, indicating phthalate/BPA modulation of GDM-related miRNAs.
Sabra et al., (2017) [13]	Spain	178 mother–infant pairs (96 AGA; 49 IUGR; 33 SGA)	Prospective case–control	Maternal & fetal serum and placental Cd, Hg, Pb, As, Zn	Not applicable	Fetal growth restriction	Small foetuses had higher maternal and fetal serum Cd than AGA; SGA foetuses showed elevated fetal Hg versus IUGR/AGA; fetal serum Cd inversely correlated with birth weight; no placental differences.
Maghbooli et al., (2018) [41]	Iran (Tehran)	100 pregnant women (50 polluted vs 50 non-polluted)	Nested case–control within cohort	Regional background PM₂.₅ and PM₁₀ (daily means for trimesters & whole pregnancy)	Placental global DNA methylation (%5-mC)	Placental methylation	First-trimester PM₂.₅ and PM₁₀ were positively associated with higher placental global DNA methylation (p=0.03 and p=0.01); S-adenosylmethionine expression also correlated with PM₂.₅ (p=0.003) and PM₁₀ (p=0.03).
Neven et al., (2018) [42]	Belgium	463 placentas	Prospective birth cohort	Trimester-specific & full-pregnancy PM₂.₅ and PM₁₀	Promoter methylation of DNA repair genes (XRCC1, hOGG1, MGMT)	Δβ per IQR increase in PM₂.₅/PM₁₀	Higher prenatal PM exposure was associated with increased promoter methylation of DNA repair genes in the placenta.
LaRocca et al., (2014) [43]	USA	179 placentas (mother–infant dyads)	Prospective birth cohort	First-trimester urinary concentrations of 11 phthalate metabolites and 8 phenols	DNA methylation at H19 DMR, IGF2 DMR0/DMR2; allele-specific expression	DMR methylation; imprinting; birth size	Higher Σphthalate and low-molecular-weight phthalate levels were associated with decreased H19 DMR methylation (p<0.05) and lower IGF2DMR0 methylation (p<0.05); ΣDEHP correlated with increased deviation in H19 allele-specific expression; no link to birth size.
Goodrich et al., (2016) [44]	South Africa	22 cord-blood plasma samples (11 south vs 11 north)	Pilot EWAS nested within the cohort	Residence in high-pollution vs low-pollution Durban and maternal HIV status	Genome-wide CpG methylation (Infinium 450K)	Differential DNA methylation patterns	Widespread hypomethylation at >430,000 CpG sites in cord blood—enriched in genes for xenobiotic metabolism, oxygen transport, and chemical stimulus perception—was linked to prenatal residency in the high-pollution area. Hypomethylation in metabolic/viral regulation pathways has also been associated with HIV.
Kaur et al., (2022) [18]	USA	563 placentas (148 RNA-Seq discovery; 415 RT-qPCR validation)	Prospective birth cohort	Maternal residential PM₂.₅ (satellite-based spatiotemporal model; trimesters & full‐pregnancy averages)	Placental mRNA of 657 lipid & glucose metabolism genes (RNA-Seq; RT-qPCR)	Differential gene expression; sex‐specific effects	Affected placental expression of 32 metabolic genes (FDR < 0.01) was linked to prenatal PM₂. Five genes (ABHD3, ATP11A, CLTCL1, ST6GALNAC4, and PSCA) were confirmed by RT-qPCR; the effects were more pronounced in male placentas, indicating sex-dependent metabolic programming.
Rudra et al., (2011) [45]	USA	3,509 women	Prospective birth cohort	Predicted ambient CO (periconceptional, trimester‐specific, last month, last 3 months) and PM₂.₅ (monthly averages)	Not applicable	Preeclampsia; preterm delivery	Periconceptional CO per 0.1 ppm was associated with a higher preeclampsia risk
Olsson et al., (2015) [46]	Sweden	100,190 singleton pregnancies	Prospective registry‐based cohort	Home‐address traffic NOₓ (pregnancy average) and vehicle flow	Not applicable	PTD; SGA; pregnancy‐induced hypertensive disorders	A 10 µg/m³ increase in pregnancy‐average NOₓ was associated with 17% higher odds of pregnancy‐induced hypertensive disorders, elevated SGA risk across NOₓ quartiles 2–4, a tendency toward increased PTD, and no association with vehicle flow.
Engström et al., (2021) [15]	Sweden	111 placentas (29 late‐onset PE; 82 controls)	Prospective case–control	First‐trimester ambient NOₓ at residence (high vs low by median split; dispersion model)	Genome-wide placental DNA methylation (EPIC array); epigenetic gestational age	DMP counts; age deceleration; maturation	Early pregnancy NOₓ exposure in late‐onset PE was linked to hypomethylation at 19 CpGs and significant epigenetic
Vrijens et al., (2020) [47]	Belgium	609 mothers and their newborns	Prospective birth cohort	Prenatal ambient air pollution exposure (PM₂.₅, black carbon) during pregnancy	Circulating maternal and cord-blood histone modifications (total histone H3, H3K4me3, H3K36me3)	Altered histone levels associated with fetal programming and adverse birth outcomes	Prenatal exposure to ambient air pollution was associated with increased circulating total histone H3 and trimethylated histones (H3K4me3 and H3K36me3) in maternal and cord blood, suggesting that air pollution may induce epigenetic alterations during fetal development.

Sample sizes ranged from small mechanistic cohorts (20 mother-newborn pairs) to large population-based registries (1,742,183 singleton births) [17,25]. Studies were conducted across North America (n = 15), Europe (n = 9), Asia (n = 7), Africa (n = 2), and Australia (n = 1) and were primarily prospective cohorts (n = 29), with five case-control or nested designs.

Eleven studies evaluated EDCs, including phthalates, bisphenol A, and phenols in maternal urine, and assessed placental or cord-blood CpG methylation [10,12,43]. Eight examined heavy metals (lead, mercury) in maternal or cord blood, focusing on global or locus-specific DNA methylation [13,29-31]. Nineteen investigated ambient air pollutants (PM₂.₅, PM₁₀, NO₂, CO, O₃, BTEX). Epigenetic endpoints predominantly involved DNA methylation (LINE-1, Alu, gene-specific CpGs via 450K/EPIC arrays), with additional assessments of mRNA expression [18,44], miRNAs [12], and histone modifications [47].

Outcomes included preterm birth, low birth weight, stillbirth, SGA, preeclampsia, and early neurodevelopment. Large epidemiological cohorts without epigenetic data were also considered for exposure-outcome context [32-34]. Together, these studies provide a comprehensive foundation for understanding how prenatal environmental exposures influence the fetal epigenome and perinatal health.

Impact of Environmental Exposure and Epigenetic Modifications on Pregnancy

Exposures such as air pollutants, heavy metals, and endocrine disruptors were consistently linked to measurable epigenetic changes in placental and fetal tissues that paralleled adverse outcomes.

Air pollution-related epigenetic alterations: Reduced global placental LINE-1 methylation with third-trimester PM₂.₅ exposure was reported in the ENVIRONAGE cohort [21], while trimester-specific NO₂ and O₃ exposures were linked to differentially methylated CpGs and DMRs in RNF39 and CYP2E1 across placenta and cord blood [20]. Increased PM₂.₅ and PM₁₀ were associated with higher promoter methylation of DNA repair genes (RCC1, hOGG1, MGMT) [42], and combined NO₂, PM₁₀, and meteorological exposures correlated with genome-wide placental CpG alterations and DMRs related to preeclampsia [27].

In high-pollution settings, extensive cord-blood hypomethylation enriched for oxygen transport and xenobiotic metabolism genes has been observed [44]. Personal BTEX exposure was associated with maternal blood hypomethylation and increased preterm birth risk [40], and early gestational NOₓ exposure was linked to placental epigenetic age deceleration in late-onset preeclampsia [15].

Air pollution and other epigenetic endpoints: Beyond DNA methylation, particulate exposure alters gene expression and histone modifications. Increased PM₂.₅ was associated with higher cord-plasma H3K4me3 and reduced H3K36me3, with similar H3K4me3 elevations observed for black carbon [47]. Prenatal PM₂.₅ exposure was also linked to altered placental expression of 32 lipid- and glucose-metabolism genes, with five (ABHD3, ATP11A, CLTCL1, ST6GALNAC4, PSCA) validated by RT-qPCR and stronger effects in males [18]. Additionally, second-trimester personal PM₁₀ exposure was associated with cord-blood methylation signatures related to cognitive outcomes at age two [26]. Collectively, these findings demonstrate that gestational particulate matter can disrupt fetal transcriptomic programs and chromatin states critical for development.

Heavy metals: Prenatal exposure to lead and mercury is associated with measurable alterations in the fetal methylome. Maternal bone lead levels were inversely correlated with cord-blood LINE-1 and Alu methylation [29], while increased second-trimester RBC-Hg was linked to higher 5-methylcytosine/5-hmC ratios and lower 5-hydroxymethylcytosine in cord blood, persisting into early infancy [31]. Broader associations between gestational metal mixtures and global 5-mC/5-hmC changes in mothers and neonates were also reported [28]. In contrast, no significant associations were observed between prenatal lead exposure and cord-blood telomere length or DNA methylation age, suggesting context-dependent effects [30].

Endocrine-disrupting chemicals: Phthalates and phenols induce sex-dependent, locus-specific epigenetic changes after crossing the placental barrier. First-trimester urinary phthalate and phenol levels were inversely associated with placental methylation at H19 and IGF2 DMRs and altered H19 allele-specific expression [43]. Maternal di-(2-ethylhexyl) phthalate metabolites were also linked to differential methylation at 25 cord-blood CpGs enriched in androgen/estrogen-response and spermatogenesis pathways [24]. Late-pregnancy phthalate exposure was associated with sex-specific placental mRNA alterations in hormone-synthesis genes (CGA, PPARG, CYP19A1, CYP11A1), modified by gestational diabetes [10]. Additionally, phthalate/BPA metabolites correlated with altered serum miRNAs, including elevated miR-9-5p, miR-29a-3p, and miR-330-3p-in gestational diabetes cases [12].

Tobacco smoke: Pregnancy-related maternal smoking causes significant methylation alterations in the cord blood. To illustrate the strong epigenomic effects of tobacco smoke, even in small cohorts, Ivorra et al. (2015) used Illumina 450K EWAS in conjunction with pyrosequencing to show global hypomethylation and specific CpG changes at AHRR and CYP1A1 in the cord blood of children born to smoking mothers [25].

Large-Scale Exposure-Outcome Studies

Thirteen population-based cohorts without epigenetic assessments consistently linked prenatal pollutant exposure to adverse birth outcomes. In China, trimester-specific exposure to PM₂, PM₁₀, SO₂, NO₂, CO, and O₃ increased stillbirth risk, particularly in the second and third trimesters [32]. A 10 µg/m³ increase in PM₂.₅ during pregnancy was associated with a 114 g reduction in birth weight, without clear preterm birth or SGA associations [33], while ozone exposure, especially at higher temperatures, was linked to increased risks of large-for-gestational-age (HR 2.16) and low birth weight (HR 1.28) [34].

Case-control and nationwide cohort studies from Finland, China, Japan, and Korea reported associations between trimester-specific pollutant exposure (including SPM >70 µg/m³, NO₂, SO₂) and preterm birth or low birth weight [17,36,37,39]. In the United States, traffic-related NOₓ was associated with a 17% increase in pregnancy-induced hypertensive disorders and elevated SGA risk [46], and periconceptional CO exposure modestly increased preeclampsia risk [45]. Collectively, these large cohorts underscore the clinical impact of prenatal environmental exposures on perinatal health.

Study Quality Assessment

Two reviewers independently evaluated each included study's quality. In the majority of the studies included in this analysis, there was a low (27 studies, 79.4%) to moderate (seven studies, 20.6%) risk of bias, showing a high percentage of positive answers to the questions of the JBI tool (Table 2).

**Table 2:** Risk of bias assessment across studies. Y: Yes; N: No; U: Unclear; N/A. Q1. Were the criteria for inclusion in the sample clearly defined? Q2. Were the study subjects and the setting described in detail? Q3. Was the exposure measured in a valid and reliable way? Q4. Were objective, standard criteria used for measurement of the condition? Q5. Were confounding factors identified? Q6. Were strategies to deal with confounding factors stated? Q7. Were the outcomes measured in a valid and reliable way? Q8. Was appropriate statistical analysis used?

Study	Q1	Q2	Q3	Q4	Q5	Q6	Q7	Q8	Yes (%)	Risk
Chen et al., [24]	Y	Y	Y	U	Y	U	Y	Y	75.0	Low
Janssen et al. [21]	Y	Y	Y	N	U	N	Y	Y	62.5	Moderate
You et al., [19]	Y	Y	Y	Y	U	N	Y	Y	75.0	Low
Ivorra et al., [25]	Y	Y	Y	Y	Y	U	Y	Y	87.5	Low
Feil et al., [26]	Y	Y	Y	Y	Y	U	Y	Y	87.5	Low
Abraham et al., [27]	Y	Y	Y	N	U	U	Y	Y	62.5	Moderate
Weyde et al., [28]	Y	Y	Y	Y	Y	U	Y	Y	87.5	Low
Pilsner et al., [29]	Y	Y	Y	Y	Y	U	Y	Y	87.5	Low
Herrera-Moreno et al., [30]	Y	Y	Y	Y	Y	U	Y	Y	87.5	Low
Cardenas et al., [31]	Y	Y	Y	Y	N	U	Y	Y	75.0	Low
Yang et al., [32]	Y	Y	Y	Y	Y	U	Y	Y	87.5	Low
Quraishi et al., [33]	Y	Y	Y	Y	Y	U	Y	Y	87.5	Low
Chen et al., [34]	Y	Y	Y	Y	N	U	Y	Y	75.0	Low
Chen et al., [35]	Y	Y	Y	N	N	N	Y	Y	62.5	Moderate
Liu et al., [36]	Y	Y	Y	Y	Y	U	Y	Y	87.5	Low
Yorifuji et al., [37]	Y	Y	Y	Y	Y	U	Y	Y	87.5	Low
Kim et al., [17]	Y	Y	Y	Y	Y	U	Y	Y	87.5	Low
Johnson et al., [38]	Y	Y	Y	Y	N	U	Y	Y	75.0	Low
Siddika et al., [39]	Y	Y	Y	Y	Y	U	Y	Y	87.5	Low
Adibi et al., [10]	Y	Y	Y	Y	Y	U	Y	Y	87.5	Low
Arbuckle et al., [11]	Y	Y	Y	Y	Y	Y	Y	Y	87.5	Low
Ladd‐Acosta et al., [20]	Y	Y	Y	N	N	U	Y	Y	62.5	Moderate
Straughen et al., [40]	Y	Y	Y	Y	Y	U	Y	Y	87.5	Low
Martínez‐Ibarra et al., [12]	Y	Y	Y	Y	Y	U	Y	Y	87.5	Low
Sabra et al. (2017) [13]	Y	Y	Y	Y	N	U	Y	Y	87.5	Low
Maghbooli et al., [41]	Y	Y	Y	Y	Y	U	Y	Y	87.5	Low
Neven et al., [42]	Y	Y	Y	Y	Y	U	Y	Y	87.5	Low
LaRocca et al., [43]	Y	Y	Y	Y	Y	U	Y	Y	87.5	Low
Goodrich et al., [44]	Y	Y	Y	Y	Y	U	Y	Y	87.5	Low
Kaur et al., [18]	Y	Y	Y	Y	Y	U	Y	Y	87.5	Low
Rudra et al., [45]	Y	Y	Y	N	N	U	Y	Y	62.5	Moderate
Olsson et al., [46]	Y	Y	Y	N	U	U	Y	Y	62.5	Moderate
Engström et al., [15]	Y	Y	U	Y	N	U	Y	Y	62.5	Moderate
Vrijens et al., [47]	Y	Y	Y	Y	Y	U	Y	Y	87.5	Low

Although quantitative synthesis was not feasible because of methodological heterogeneity, the included studies consistently demonstrated directional associations between prenatal environmental exposures, epigenetic dysregulation, placental dysfunction, and adverse perinatal outcomes.

Discussion

This systematic review synthesizes evidence from 34 human studies demonstrating consistent associations between prenatal exposure to ambient air pollutants, heavy metals, EDCs, and tobacco smoke and epigenetic alterations in placental, cord-blood, and maternal tissues. Across studies, environmentally induced changes in DNA methylation, histone modifications, and non-coding RNA expression frequently paralleled increased risks of preterm birth, low birth weight, hypertensive disorders of pregnancy, gestational diabetes, stillbirth, and early neurodevelopmental alterations. Despite methodological heterogeneity, the findings collectively support the role of epigenetic dysregulation as a biologically plausible mediator linking prenatal environmental exposure to adverse perinatal outcomes.

Main Findings and Comparison with Existing Literature

Prenatal environmental exposures can reshape the developing fetal epigenome, potentially influencing disease susceptibility across the life course [7,41,48]. This systematic review synthesizes evidence from 34 human studies (2009-2024) demonstrating consistent associations between prenatal exposure to ambient air pollutants, heavy metals, EDCs, and tobacco smoke and epigenetic alterations in placental, cord-blood, and maternal tissues. These molecular changes frequently parallel increased risks of preterm birth, low birth weight, hypertensive disorders of pregnancy, gestational diabetes, and impaired neurodevelopment.

Air pollution exposures, including NO₂, O₃, CO, PM₂.₅/PM₁₀, and BTEX, were consistently associated with both global and gene-specific DNA methylation changes. Reduced LINE-1 methylation in relation to PM₂.₅ [21] and DMRs in RNF39 and CYP2E1 associated with NO₂/O₃ exposure [20] illustrate reproducible locus-specific effects across cohorts. Altered placental expression of metabolic genes following PM₂.₅ exposure [18] and histone mark perturbations such as H3K4me3 and H3K36me3 [47] further indicate that air pollution exerts multi-layered epigenetic influence. The consistency of findings across diverse geographic populations strengthens causal inference.

Our findings extend earlier syntheses linking environmental exposures to epigenetic dysregulation. Initial reviews connected air pollution to global and candidate-gene methylation changes [49], while more recent analyses evaluated epigenetic regulation of environmental influences on child health [50]. However, many prior reviews did not integrate in utero exposures with pregnancy-specific outcomes. By incorporating multiple exposure classes and linking epigenetic alterations to clinically defined perinatal outcomes, the present review provides a more integrated epidemiological-mechanistic perspective.

Evidence for EDCs supports sex-specific and locus-specific epigenetic effects, particularly at imprinting control regions such as H19/IGF2 [25,51]. Associations between maternal phthalate exposure and altered placental mRNA expression [10], genome-wide CpG methylation changes [24], and dysregulated serum miRNAs in gestational diabetes [12] suggest endocrine-mediated epigenetic reprogramming. Similarly, heavy metal exposures were associated with global hypomethylation and altered 5mC/5hmC balance [28,29,31], reinforcing prior observations of metal-induced epigenetic instability [1,52]. Tobacco smoke exposure demonstrated robust and reproducible cord-blood methylation signatures, including global hypomethylation and locus-specific changes at AHRR and CYP1A1 [25,53], underscoring its strong epigenomic imprint.

Mechanistic Pathways and Epigenetic Programming

Across exposure classes, convergent biological mechanisms are plausible. Air pollutants may induce oxidative stress and inflammatory signaling that activate DNA methyltransferases and histone-modifying enzymes. EDCs can disrupt hormone-responsive gene networks and imprinting control regions, whereas metals such as Pb²⁺ and Hg²⁺ may interfere with ten-eleven translocation enzymes, altering the balance between 5-methylcytosine and 5-hydroxymethylcytosine. These pathways provide mechanistic plausibility for observed associations with fetal growth restriction, preterm birth, and hypertensive disorders of pregnancy. Emerging evidence further suggests that environmental exposures may interact with psychosocial stress, inflammatory signaling, endocrine pathways, and placental dysfunction within a broader gestational exposome framework. Shared mechanisms involving oxidative stress, cytokine activation, and altered neuroendocrine regulation may contribute to fetal programming and subsequent neurodevelopmental vulnerability. Although these dimensions were not extensively evaluated in the included studies, they represent important areas for future maternal and fetal epigenetic research.

The findings of this review also align with emerging gestational exposome models suggesting that environmental pollutants may interact with psychosocial stress, inflammatory pathways, endocrine signaling, and placental programming to influence fetal development. Although neuroepigenetic and perinatal mental health outcomes were not extensively evaluated in the included studies, growing evidence supports the role of combined environmental and psychosocial exposures in shaping long-term neurodevelopmental trajectories.

Strengths and Limitations

This systematic review had several limitations. Considerable heterogeneity existed across included studies regarding exposure assessment, biological tissues, epigenetic methodologies, gestational timing, and clinical outcome definitions, limiting direct comparability and preventing formal meta-analysis. Residual confounding and variability in environmental exposure characterization may also have influenced reported associations. In addition, emerging exposures such as microplastics, climate-related stressors, and psychosocial determinants remain underrepresented in the current evidence base. A key strength of this review lies in its integration of multiple exposure categories, epigenetic layers (DNA methylation, histone modifications, non-coding RNAs), and clinically relevant outcomes across diverse populations. Future studies should incorporate longitudinal exposure assessment, multi-omics approaches, and functional validation to clarify causal pathways and identify modifiable windows of vulnerability.

Clinical and Public Health Implications

The findings of this review reinforce the importance of minimizing maternal exposure to environmental pollutants during pregnancy through improved environmental monitoring, public health awareness, and preventive strategies. Identification of environmentally responsive epigenetic biomarkers may also support future risk stratification and early intervention approaches in maternal and fetal medicine.

## Conclusions

Prenatal environmental exposures, including air pollutants, heavy metals, endocrine disruptors, and tobacco smoke, are consistently associated with epigenetic alterations in fetal tissues that parallel increased risks of adverse birth outcomes. These findings underscore the importance of reducing maternal pollutant exposure and support the future integration of epigenetic biomarkers, environmental surveillance, and preventive maternal and fetal health strategies. Further longitudinal studies incorporating combined environmental exposures, psychosocial stressors, neurodevelopmental outcomes, and multi-omics approaches are needed to better understand long-term intergenerational health effects.
